# Conductive proteins‐based extracellular electron transfer of electroactive microorganisms

**DOI:** 10.1002/qub2.24

**Published:** 2023-11-27

**Authors:** Junqi Zhang, Zixuan You, Dingyuan Liu, Rui Tang, Chao Zhao, Yingxiu Cao, Feng Li, Hao Song

**Affiliations:** ^1^ Frontier Science Center for Synthetic Biology (Ministry of Education), Key Laboratory of Systems Bioengineering, and School of Chemical Engineering and Technology Tianjin University Tianjin China

**Keywords:** *c*‐type cytochromes, conductive nanowires, extracellular electron transfer, kinetic parameters, synthetic biology

## Abstract

Electroactive microorganisms (EAMs) could utilize extracellular electron transfer (EET) pathways to exchange electrons and energy with their external surroundings. Conductive cytochrome proteins and nanowires play crucial roles in controlling electron transfer rate from cytosol to extracellular electrode. Many previous studies elucidated how the *c*‐type cytochrome proteins and conductive nanowires are synthesized, assembled, and engineered to manipulate the EET rate, and quantified the kinetic processes of electron generation and EET. Here, we firstly overview the electron transfer pathways of EAMs and quantify the kinetic parameters that dictating intracellular electron production and EET. Secondly, we systematically review the structure, conductivity mechanisms, and engineering strategies to manipulate conductive cytochromes and nanowire in EAMs. Lastly, we outlook potential directions for future research in cytochromes and conductive nanowires for enhanced electron transfer. This article reviews the quantitative kinetics of intracellular electron production and EET, and the contribution of engineered *c*‐type cytochromes and conductive nanowire in enhancing the EET rate, which lay the foundation for enhancing electron transfer capacity of EAMs.

## 1 INTRODUCTION

Electroactive microorganisms (EAMs) enable transfer of electrons to extracellular insoluble electron acceptors, such as metal oxides and electrodes via electron conduit that is composed of multi‐heme conductive cytochromes and the self‐synthesized and secreted redox‐active compounds [1, 2]. EAMs could act as biocatalyst, enabling many bio‐electrochemical systems and their applications, such as microbial fuel cells for environmental remediation and electricity harvest, microbial electrosynthesis for CO_2_ reduction and value‐added chemicals production, and microelectronic devices for environmental sensing [3, 4, 5, 6, 7]. However, the low electron transfer capacity of EAMs dramatically limits their practical applications [2, 8].

In the last decades, with the development of synthetic biology and microbial electrochemistry, theoretical calculations combined with engineering strategies to improve electron transfer rate attracted extensive attention [9, 10, 11, 12, 13]. Mathematical modeling to estimate kinetic parameters of intracellular electron production as well as extracellular electron transfer (EET) kinetic parameters provides rational guidance in the design and engineering of EAMs. For example, by building a theoretical model in *Geobacter sulfurreducens*, Bonanni et al. found that electron transfer from acetate to inner cytochromes was a major limitation in current production, which suggested that enhancing the expression level of cytochromes may be an important strategy to overcome the low electron transfer capacity of EAMs [14]. Actually, EAMs contain abundant *c*‐type cytochromes (*c*‐Cyts) and conductive nanowires (i.e., e‐pili). *c*‐Cyts are formed by multiple heme cofactors covalently bound to the *c*‐Cyt protein precursors via thioether bonds, acting as electron carrier for near‐range electron transfer [15]. Conductive e‐pili (i.e., the type IV pilin PilA in *Geobacter*) consists of extracellular protein filaments, which connect the inner membrane to extracellular terminal electron acceptors, thus performing long‐distance electron transfer [16, 17, 18]. Up to now, although studies have shown that enhanced cytochrome expression or synthesis of conductive pili can promote EET, how cytochromes and pili are assembled and endowed with conductive properties are remain poorly understood.

In this review, with the focus on *c*‐Cyts and conductive nanowire of EAMs, we firstly quantify the kinetic parameter of intracellular electron production and EET, then discuss the structural components, functions, and engineering strategies of cytochromes and conductive nanowires. Lastly, we identify the shortcomings and future research needs to boost EET rate by enhancing the conductivity and expression of *c*‐Cyts and conductive nanowire.

## 2 QUANTITATIVE ELECTRON TRANSFER KINETICS OF EAMs

**FIGURE 1:**
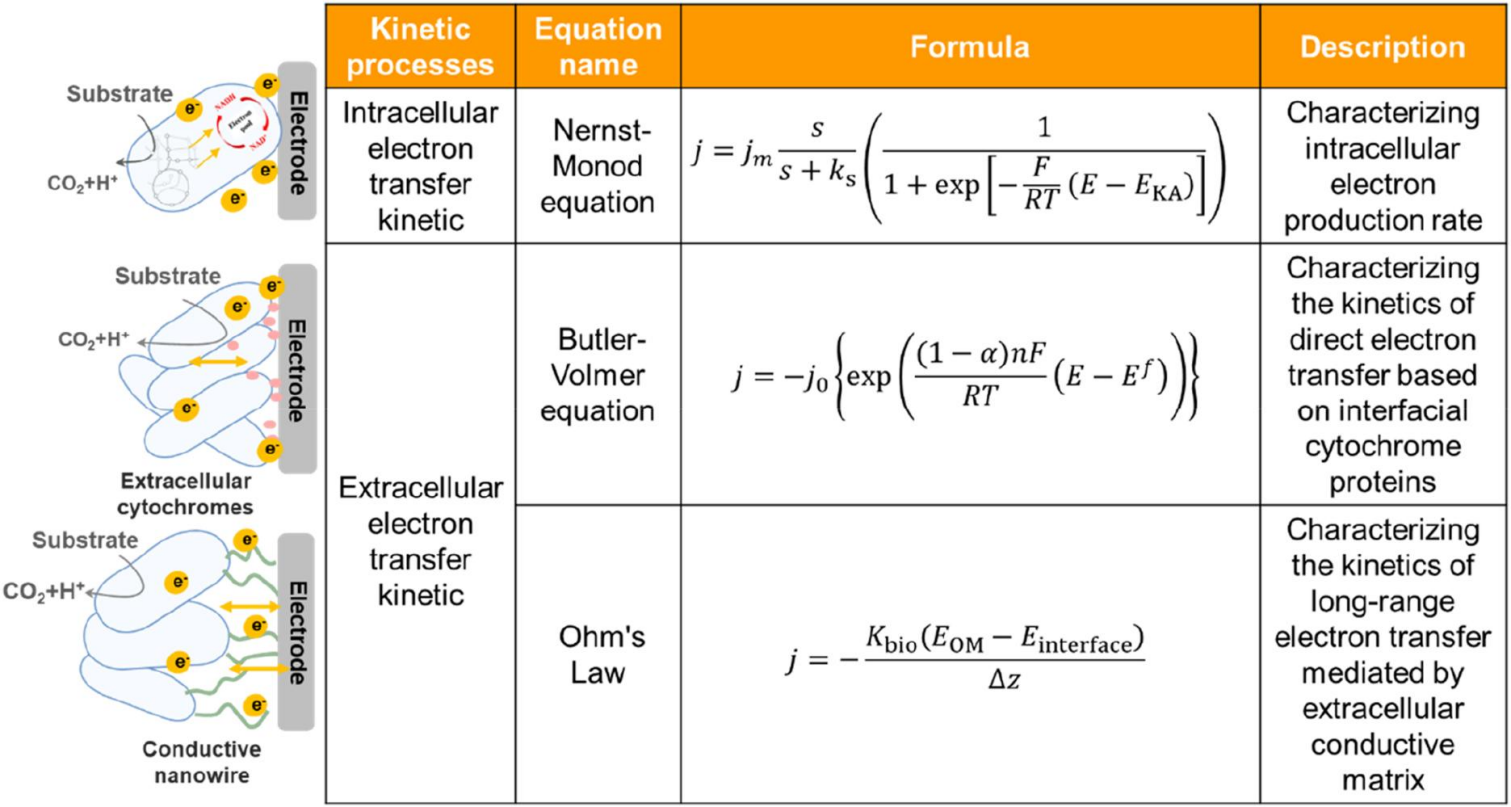
DET kinetics of EAMs. DET, direct electron transfer; EAMs, electroactive microorganisms.

Electron transfer capacity is necessary for microorganisms to maintain their redox state by respiration under anaerobic conditions. However, the inefficient electron transfer capacity severely limits the application potential of EAMs. Therefore, it is crucial to further improve the electron transfer capability by mathematical modeling for resolving the EET kinetic parameters. In this section, we quantify the electron transfer kinetics for intracellular electron production and EET (Figure 1). The intracellular electron transfer from donor to anode can be described by the Nernst–Monod equation, while the EET can be described by the Butler–Volmer equation. In addition, the electron transfer and energy loss in extracellular matrix can be characterized by the metal‐like conduction equation [19].

### 2.1 Intracellular electron production kinetic parameters

In an electrocatalytic system, once the microorganism metabolizes the substrate to generate electrons, the electron transfer system will be directly initiated, prompting electrons to reach the extracellular cytochromes along the electron transfer pathway. The substrate utilization rate of microorganisms can be represented by the Monod equation. The capacity of the substrate to be converted into electrons directly determines the catalytic activity of electrocatalytic systems. This process can be quantified by the Nernst–Monod equation (Equation 1) [20].

The Nernst–Monod equation is derived from the Monod equation and can be used to describe how the current generation is correlated with the anode potential and reactant concentration in an electrochemical reaction:

(1) $$j=j_{\max}\;\frac{s}{s+k_{s}}\left(\frac{1}{1+\exp\left[-\frac{F}{RT}\;\left(E-E_{\text{KA}}\right)\right]}\right)$$

where, *j* represents the actual current density obtained by EAMs, *j*_max_ is the theoretical maximum current density. *S* is the substrate concentration in the liquid, and *K_s_* represent the apparent half‐saturation concentration of the substrate in the biofilm. *R* is the ideal gas constant (8.3145 J mol^−1^ K^−1^), and *F* is the Faraday constant (96,485 C mol^−1^ e^−^). *T* is the temperature (K), and *E*_KA_ is the anode potential at which the current is half the maximum current (V). *E* is the anode potential (V).

The Nernst–Monod Equation (1) showed that the ability of EAMs to generate current is closely related to the anode potential and the substrate concentration. In addition, the Nernst–Monod equation can also be used to model the response of EAMs to the change of anode potential [21]. In the Equation (1), when *E*_KA_ = *E*, the exponential term is equal to 1 and the actual current density obtained is $\frac{1}{2}\;j_{\max}$. When *E*_KA_ is determined, the rate of substrate utilization increases as the anode potential *E* increases. However, when the *E* is sufficiently high, the rate of substrate utilization will be limited due to the anode electron acceptor saturates and donor oxidation [22].

### 2.2 Extracellular electron transfer kinetic parameters

Although the Nernst–Monod Equation (1) describes the relationship between the intracellular electron generation rate and the anode potential, it does not reflect the intracellular electron transfer mechanism. The Butler–Volmer equation is of use to model electron transfer mechanisms [20]. For example, in the electron transfer process, cytochromes on the cell surface can directly contact anode for electron transfer, which effectively reduces the loss of extracellular potential. However, due to the fact that the electron transfer of cytochromes is reversible, it is speculated that the process may also be reversible. Therefore, the Butler–Volmer equation (Equation 2) can be used to describe the rate change of the reversible reaction occurring at the cell‐electrode interface [23].

(2) $$j=j_{0}\left\{\exp\left(\frac{\left(1-\alpha \right)nF}{RT}\left(E-E^{f}\right)\right)\right\}$$

where *E* is the anode potential (V), *E* ^*f*^ is the standard potential (V), and *j*_*0*_ is the exchange current density (Am^−2^). *α* is the electron‐transfer coefficient of the anodic reaction. In Equation (2), if the interfacial electron transfer is reversible, the value of *j* will directly depend on the difference in electrochemical potential between the anode (*E*) and the electrochemically active species (*E* ^*f*^).

Additionally, in the electron transfer process, cytochromes, and conductive nanowire on the surface of EAMs, together with extracellular mucins and polysaccharides, form the extracellular matrix that mediates the electron transfer process between cells and electrodes. Due to the properties of extracellular polymeric substances, the extracellular matrix may act as a semiconductor rather than a conductor. As a result, this electron transfer process can be simulated using the Ohm’s Law (Equation 3) [22]:

(3) $$j\;=\;-\frac{K_{\text{bio}}\left(E_{\text{OM}}-E_{\text{interface}}\right)}{∆Z}$$

where *K*_bio_ correlates with the conductivity of the extracellular matrix, containing cytochromes and pili (R^−1^ L^−1^). *E*_OM_–*E*_interface_ is extracellular potential losses. In this equation, the higher conductivity of the extracellular matrix is negatively correlated with the current density. A higher *K*_bio_ can reduce the extracellular potential losses (*E*_OM_ – *E*_interface_).

In summary, these mathematical models allow for numerical estimation of the kinetic transfer process of electrons from substrate to electrodes, providing theoretical support to explore the in‐depth electron transfer mechanism and being an important tool to guide the engineering of EAMs. However, these mathematical models have essentially been developed under ideal conditions. It remains challenging for a comprehensive analysis of energy conversion and electron transfer in EAMs. Therefore, theoretical models should be integrated with synthetic biology technologies to further elucidate the kinetic and thermodynamic mechanisms of complex electron transfer processes.

## 3 STRUCTURE, FUNCTION, CONDUCTIVITY MECHANISM, AND EXPRESSION OF CYTOCHROMES

It has been widely recognized that multi‐heme cytochromes play an essential role in mediating intracellular electrons across the cell envelope [24]. In this section, we focus on the structure, assembly, and conductivity mechanism of cytochromes, and discuss the effects of enhancing cytochrome expression on EET.

### 3.1 Structures and functions of cytochromes

Many microorganisms rely on the electron transfer function of cytochromes to oxidize external substances to enable respiration. These cytochromes form an electron transport pathway originating from the inner membrane, spanning the periplasm, and outer membrane, and extending into the extracellular environment. Here, we classified the cytochromes into three types according to their positions in the cell membrane and the electron transfer pathway: inner membrane, periplasmic space, and outer membrane cytochromes. A description of the structures and functions of the representative cytochromes of each category is presented.

#### 3.1.1 Inner membrane cytochromes

**FIGURE 2:**
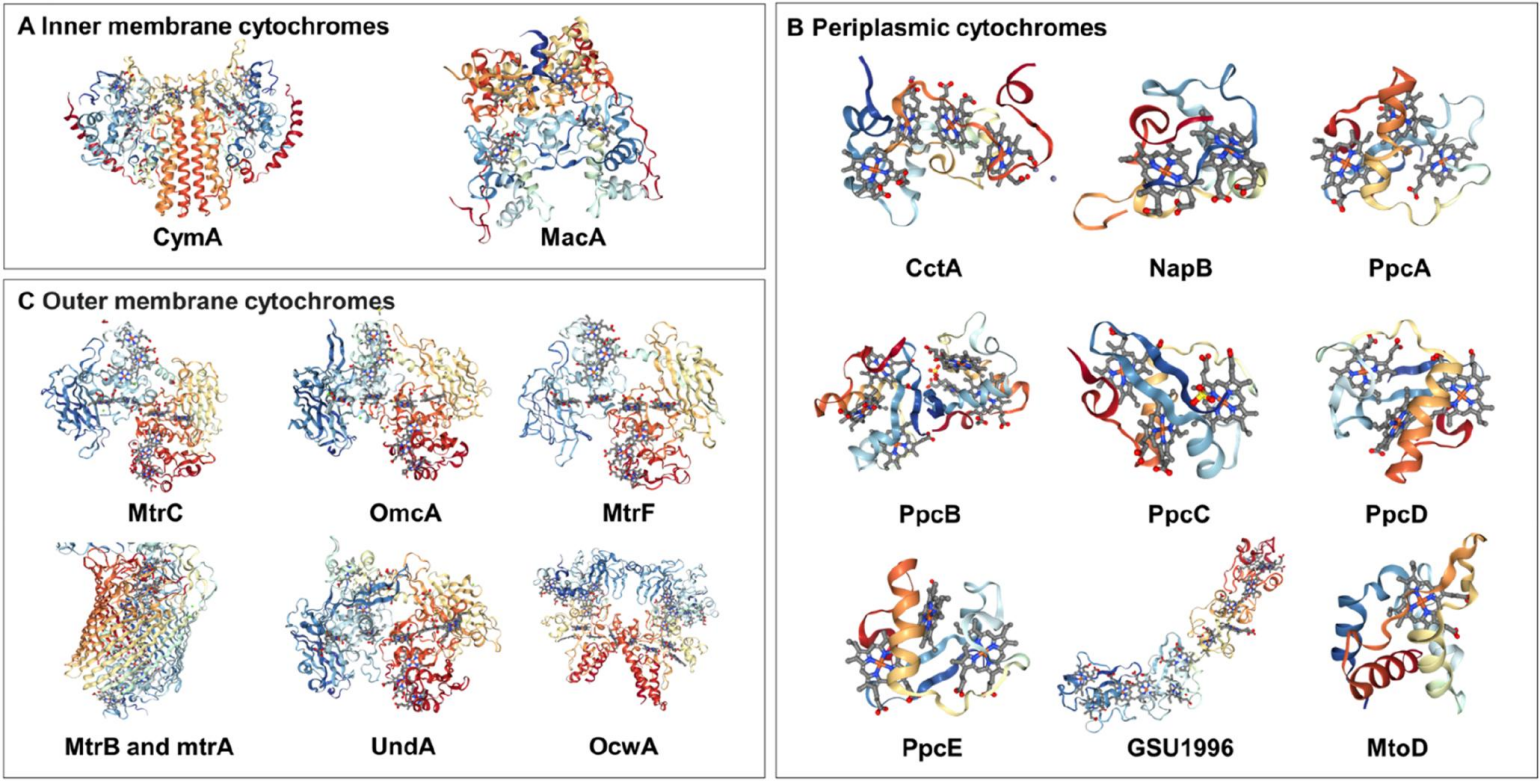
Structure of *c*‐Cyts in the EET pathway of EAMs. Electron‐transferring *c*‐Cyts contain several hemes that are electron transfer centers composed of porphyrin rings and iron atoms. (A) The structure of inner membrane cytochromes. CymA is from *Shewanella oneidensis* and MacA is from *Geobacter sulfurreducens.* (B) The structure of periplasmic cytochromes. CctA and NapB are from *S. oneidensis*. PpcA‐PpcE and GSU1966 are from *G. sulfurreducens.* MtoD is from *Sideroxydans lithotrophicus* ES‐1. (C) The structure of outer membrane cytochromes. MtrABC complex, OmcA, MtrF, and UndA are from *S. oneidensis.* OcwA is from the Gram‐positive bacterium *Thermincola potens.* All protein structures from Protein Data Bank (PDB). *c*‐Cyts, *c*‐type cytochromes; EAMs, electroactive microorganisms; EET, extracellular electron transfer.

The inner membrane cytochromes are important mediators linking intracellular electron donors to extracellular electron acceptors (Figure 2A). The *c*‐type cytochrome CymA (tetrahaem cytochrome *c*) anchored in the inner membrane of *Shewanella oneidensis* MR‐1 and *Sideroxydans lithotrophicus* is an important quinol dehydrogenase, which can oxidize quinol and transfer the released electrons to the periplasmic cytochromes [25, 26]. This CymA protein belongs to the NapC/NirT superfamily and contains three low‐spin bis‐histidine coordinated hemes and one high‐spin heme coordinated with His/H_2_O, where the high‐spin heme is adjacent to the quinone binding site and allows to directly capture electron [25, 27]. Similarly, inner membrane diheme cytochrome MacA from *G. sulfurreducens* exhibits a similar function to CymA, who contains two low‐spin heme groups axially coordinated by two histidine residues (His‐His) and can capture electrons from the quinone/menaquinone pool and reduces the periplasmic triheme cytochrome PpcA [28]. Notably, the molecular weights of CymA and MacA are 20.8 and 41.8 kDa, respectively, and the latter only contains two hemes in its protein structure. It is needed to further elucidate whether there is difference in the electron transfer efficiency between the two proteins.

CbcL and ImcH, which are present in the inner membrane of *G. sulfurreducens*, also have the similar function as CymA and MacA [29, 30]. CbcL contains nine heme, of which di‐heme is located in the transmembrane structural domain, and the other six homes are located in the soluble periplasmic domain that can link to a periplasmic cytochrome PpcA [31]. While ImcH is another inner membrane heptaheme cytochrome, containing three transmembrane helices that are efficiently anchored to the cell membrane [29]. However, CbcL and ImcH may operate only when exposed to low redox potentials. Additionally, MtoC, an inner membrane *c*‐Cyt from *Gallionella capsiferriformans* ES‐2 (ES‐2) and *Dechloromonas aromatica* RCB (RCB), fulfills a similar function of CymA that can reduce quinone (Q) to quinol (QH_2_) [32].

In conclusion, there are a small number of inner membrane cytochromes reported in EAMs. These proteins receive electrons from the quinone pool and deliver electrons to peripheral cytochromes, allowing electrons to be eventually transported to the outer membrane cytochromes.

#### 3.1.2 Periplasmic cytochromes

In general, outer membrane cytochromes often require periplasmic cytochromes to provide electrons for efficient electron transfer (Figure 2B). Periplasmic cytochrome content accounts for approximately 60% of total cytochromes in EAMs. For example, the periplasm of *S. oneidensis* MR‐1 contains abundant cytochromes, most abundantly FccA, NrfA, NapB, and CctA (STC), which can accept electrons from the intramembrane cytochrome CymA and deliver electrons to the Mtr or DmsEFAB complex [33, 34, 35]. In these periplasmic cytochromes, FccA is the unique soluble periplasmic tetraheme flavocytochrome *c* that contains three domains: a heme domain, a flavin domain and a clamp domain. FccA can engage in specific recognition and docking bind to CymA via heme II. CctA is a tetraheme cytochrome containing four hemes in a double histidine axial coordination [36]. Two other cytochromes, SirA and Octaheme Tetrathionate Reductase, associated with cellular sulphite reduction and nitrite reduction, were also found in the periplasm of *S. oneidensis* MR‐1 [37, 38, 39].

A considerable amount of cytochromes, including PpcA‐PpcE and GSU1996, is also present in the periplasmic space of *Geobacter sulphurreducens* PCA. PpcA can work with its family proteins PpcB‐PpcE to complete electron transfer from the inner to the outer membrane [40, 41, 42]. These periplasmic cytochromes of the PpcA family are smaller proteins of about 10 kDa and contain three low‐spin heme groups with His‐His axial ligands. The dodecahaem cytochrome GSU1996 (42.3 kDa) is composed of four similar triheme structural domains, connected by a flexible connector, where each structural domain is present as a low‐spin heme containing His‐His and His‐Met axial coordination. Within each structural domain, two heme have a His‐His axial coordination, while the third consists of a histidine and a methionine residue (His‐Met) axially coordinated [43]. Moreover, researchers also identified cytochrome GSU0105 with a greater range of redox potentials than the PpcA family in *G. sulfurreducens*. This cytochrome has different amino acid sequence and spatial structure in comparison to the cytochromes from the PpcA family. Analysis of the protein structure of GSU0105 revealed a distinct axial coordination of the heme and a more pronounced reduction potential value of the protein, suggesting that GSU0105 has the potential to transfer electrons on a larger scale [44]. Recently, a novel periplasmic cytochrome GSU2515 was also characterized. This periplasmic cytochrome is a low‐spin monoheme cytochrome with a disordered N‐terminal region and a α‐helical C‐terminal structural domain containing a heme group. Based on this unique structure, GSU2515 can form a redox complex with PccH for electron capture and transport [45]. In *S. lithotrophicus* ES‐1, a periplasmic cytochrome mtoD (monoheim *c*‐Cyt) with the ability to transfer electrons from the outer membrane cytochrome MtoA to the inner cell membrane CymA was identified [26].

In summary, EAMs are rich in periplasmic cytochromes, which play an important bridging role in electron transfer across inner and outer membranes. However, the structure and function of a large number of periplasmic cytochromes remain unresolved.

#### 3.1.3 Outer membrane cytochromes

Outer membrane cytochromes are important mediators that facilitate rapid electron transfer between EAMs and electron acceptors. Depending on the distribution of cytochrome protein, the outer membrane cytochromes can be subdivided into pore protein‐cytochrome complexes and cell surface‐attached cytochromes (Figure 2C). These cytochromes form an electron transfer channel that receives electrons from the periplasmic cytochromes and eventually transfers them to the extracellular acceptor.

The pore protein‐cytochrome complex usually consists of a trans‐epithelial β‐barrel wrapped around one or more cytochrome proteins, with electron transfer via the heme in the cytochrome. Among them, the MtrCAB complex from *S. oneidensis* MR‐1 is one of the most intensively studied [46]. The crystal structure showed that the MtrB protein consists of 26 inversely parallel β‐folded strands and outwardly extending loops that span the outer cell membrane, forming a 30 Å pore size into which both MtrC and MtrA are partially inserted [46, 47]. In this complex, electrons mainly transfer from the periplasm to the outer terminal electron acceptor via 20 heme at a distance of 185 Å [48, 49]. Moreover, MtrDEF complex, DmsEFAB and SO4362‐SO4357 are also highly homologous to the MtrCAB complex of *S. oneidensis* MR‐1 [50, 51]. However, the crystal structure of these protein complexes has not been fully resolved. There are two outer membrane pore‐cytochrome complexes similar to MtrCAB in *G. sulfurreducens* PCA, including OmaB‐OmbB‐OmcB complex and OmaC‐OmbC‐OmcC complex. Both OmaB and OmaC proteins are octahaem periplasmic proteins, OmbB/OmbC are transmembrane pore‐porins and OmcB/OmcC are dodecameric extracellular cytochromes located in the extracellular membrane [52]. In addition to *S. oneidensis* MR‐1 and *G. sulfurreducens* PCA, pore protein‐cytochrome complexes were found in *Rhodopseudomonas palustris* TIE‐1, *S*. *lithotrophicus* ES‐1, *Acidithiobacillus ferrooxidans*, *Aeromonas hydrophila*, *Ferrimonas balearica*, *Rhodoferax ferrireducens*, and other strains [26, 32, 52, 53, 54, 55, 56, 57, 58, 59].

Although the pore protein‐cytochrome complex is the most important pathway for electron transport, cell surface‐attached cytochromes also play a similar function in electron transfer, especially in the interfacial electron transfer. In the case of *S. oneidensis* MR‐1, for example, the outer membrane attached OmcA protein acts as a terminal reductase and is responsible for EET [60]. Observation of the structure of OmcA revealed that there are four structural domains, of which structural domain I and domain III contain a Greek key split‐barrel structure, while structural domains II and IV can covalently bind covalently to five heme. In practical electron transfer, two OmcA cytochromes can interact to form a dimer near heme five of structural domain II, allowing for long distance electron transfer [24]. In *G. sulfurreducens* PCA, there are six cytochromes attached to the cell surface: OmcF, OmcT, OmcS, OmcE, OmcZ, and PgcA, which are more loosely bound to the cell surface [61, 62, 63, 64, 65]. These cytochrome proteins contain multiple‐heme, which are arranged orderly in polymeric assemblies, forming extracellular conductive nanowires and allowing long distance electron transfer. Moreover, in the Gram‐positive bacterium *Thermincola potens*, the cytochrome TherJR_2595 (OcwA, outer cell wall protein A), attached to the outer surface of the cell, was also found to interact with the cytochrome proteins TherJR_1122, TherJR_0333, and TherJR_1117 and work together to enable electron transfer [66].

Collectively, the abundance of cytochrome proteins of EAMs collaborates to build a complex network of electron transport, effectively connecting the cell to the outside environment for electron exchange. However, the functions and structures of a significant proportion of cytochrome proteins have not yet been experimentally demonstrated, thereby allowing for in‐depth structural elucidation by combining artificial intelligence and machine learning [67, 68].

### 3.2 Conductive mechanisms of cytochromes

**FIGURE 3:**
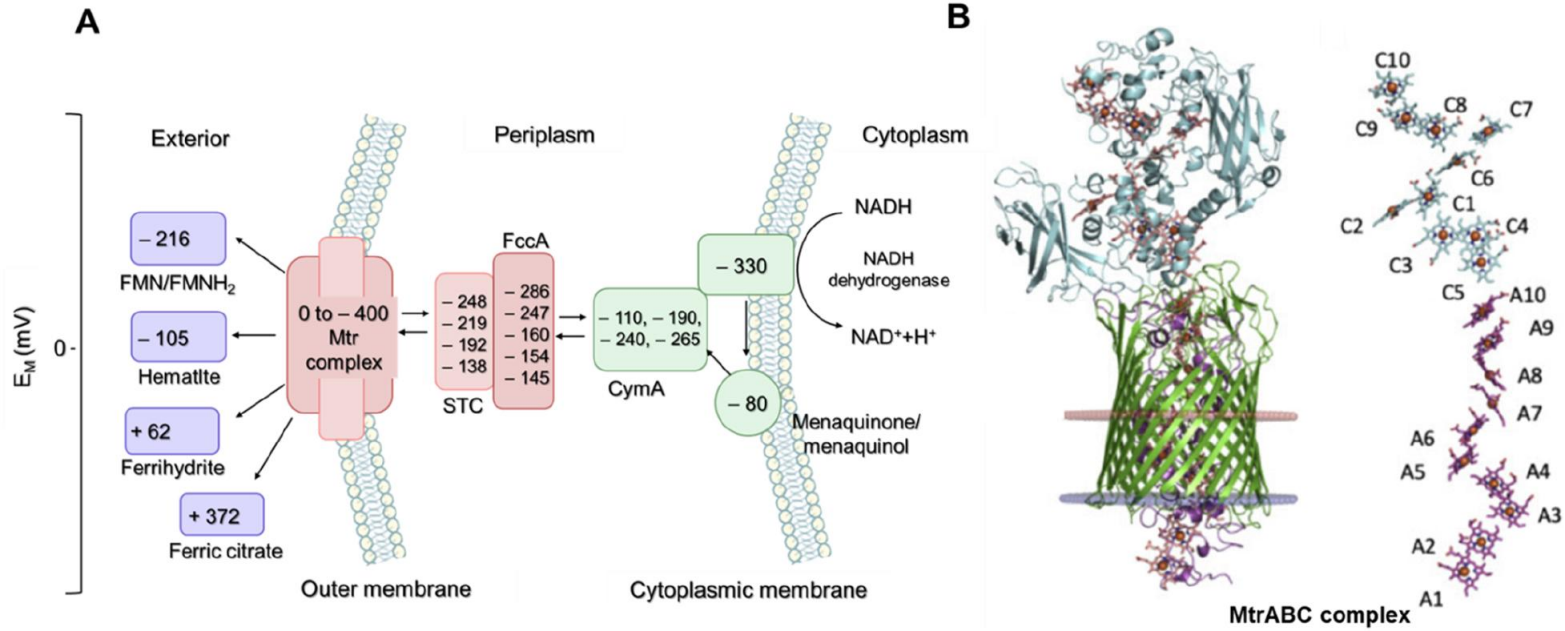
Conductive mechanisms of cytochromes. (A) The reduction potentials (vs. SHE at pH = 7) of diverse cytochromes in electron transport pathway was shown [47]. (The redox potential of CymA ranges from −110 to −265, FccA from −145 to −286, STC from −138 to −248, and the Mtr complex redox potential has a broader range of 0 to −400). (B) Mechanism of electron transfer in the crystal structure of the MtrABC complex. The crystal structure consists of MtrA (magenta), MtrC (blue) and β‐barrel protein MtrB (green). MtrA has a rod‐like structure with a length of 104 Å, it was sheathed inside MtrB with the N‐terminal protruding from the periplasmic side, and the C‐terminus in contact with MtrC (left). The heme 5 of MtrC (C5) can interact with the heme 10 of the MtrA (A10) to form a 20‐heme chain with a length of 185 Å, allowing electrons to move across the heme network by hopping (right) [24, 47].

The cytochrome‐mediated electron transfer mode is an important channel for energy exchange between cells and the external environment. Understanding this electron transfer mechanism usually requires combining the 3D structure of the protein with thermodynamic and kinetic parameters [69]. The rate of cytochrome protein‐mediated electron transfer is closely related to the spatial arrangement of hemes within the protein as well as the reduction potential. The wide range of reduction potential of heme directly confers different reduction potentials to cytochrome proteins. The reduction potentials of cytochromes associated with the EET pathway is shown in Figure 3A.

At the macroscopic level, the reduction potential is susceptible to the influence of the surrounding electron flux. For example, thermodynamic and kinetic data suggest that in the FccA protein, when low electron fluxes are present, FccA cannot be completely reduced and instead transfers electrons to the outer membrane reductases, such as omcA and mtrC [70]. Furthermore, it was also found that the reduction potentials of the 20 hemes in the *S. oneidensis* Mtr complex range from approximately 0 to −400 mV (vs. SHE). With such a wide range of reduction potential, the MtrCAB complex can accept electrons from CymA via the periplasmic cytochrome CctA or FccA and eventually transfer electrons to extracellular electron acceptors [47].

At the microscopic level, we discuss the electron transfer mechanism between cytochromes by taking an example of the MtrCAB complex of *S. oneidensis*. In the MtrCAB complex, MtrC contains 10 hemes distributed in structural domains II and IV and arranged in a specific rule to form a central core, while structural domains I and III flank either side [24]. The heme 10 of MtrC is exposed to the extracellular environment and can transfer electrons directly to electron acceptors, while the exposed heme 5 can interact with the heme 10 of the MtrA to form a 20‐heme chain, achieving a long‐distance hopping of electrons. MtrA has a rod‐like structure, with a length of 104 Å [71]. The adjacent heme pairs in the MtrA are arranged in alternating parallel and perpendicular ring planes. The distance from heme 1 to heme 10 is 80 Å in MtrA, while the distance from heme 1 to the periplasm is 20 Å. The 20 Å distance exposed in the periplasm space allows for direct capture of electrons from the periplasmic cytochromes and rapidly transfer electrons to MtrC. Moreover, MtrA has a maximum cross‐section of elliptical cross‐section (25 × 50 Å), which facilitates its insertion into the hydrophobic barrel protein MtrB, consisting of 26 inversely parallel β strands, to form the MtrAB complex. After MtrA is inserted into MtrB, it can be specifically oriented, allowing the heme chain to be perpendicular to the cell membrane, which eventually, allows MtrC to use residues around heme 5 to form hydrogen bond with MtrAB and form a stable Mtr complex (Figure 3B) [47].

In conclusion, the electron transfer capacity of EAMs depends on the redox potential of cytochromes inside and outside of the cell and the heme spatial distribution. In fact, the redox potential of proteins is defined by each individual heme [72]. Moreover, the spatial distribution of hemes in the cytochrome protein also determines the electron transfer rate, but how fast this electron transfer rate is requires more careful simulations and experiments to confirm [47].

### 3.3 Enhancing the expression of cytochromes

3.3.1

Cytochromes are an important component of energy exchange between EAMs and the extracellular environment. Therefore, enhancing the expression of cytochromes to boost electron transport has received extensive attention. Here, we summarize the engineered strategies from three aspects: enhancing the expression of the inner membrane cytochromes, and the outer membrane cytochromes expression; and optimizing the outer membrane cytochromes expression.

#### 3.3.2 Enhancing the expression level of inner membrane cytochromes

**FIGURE 4:**
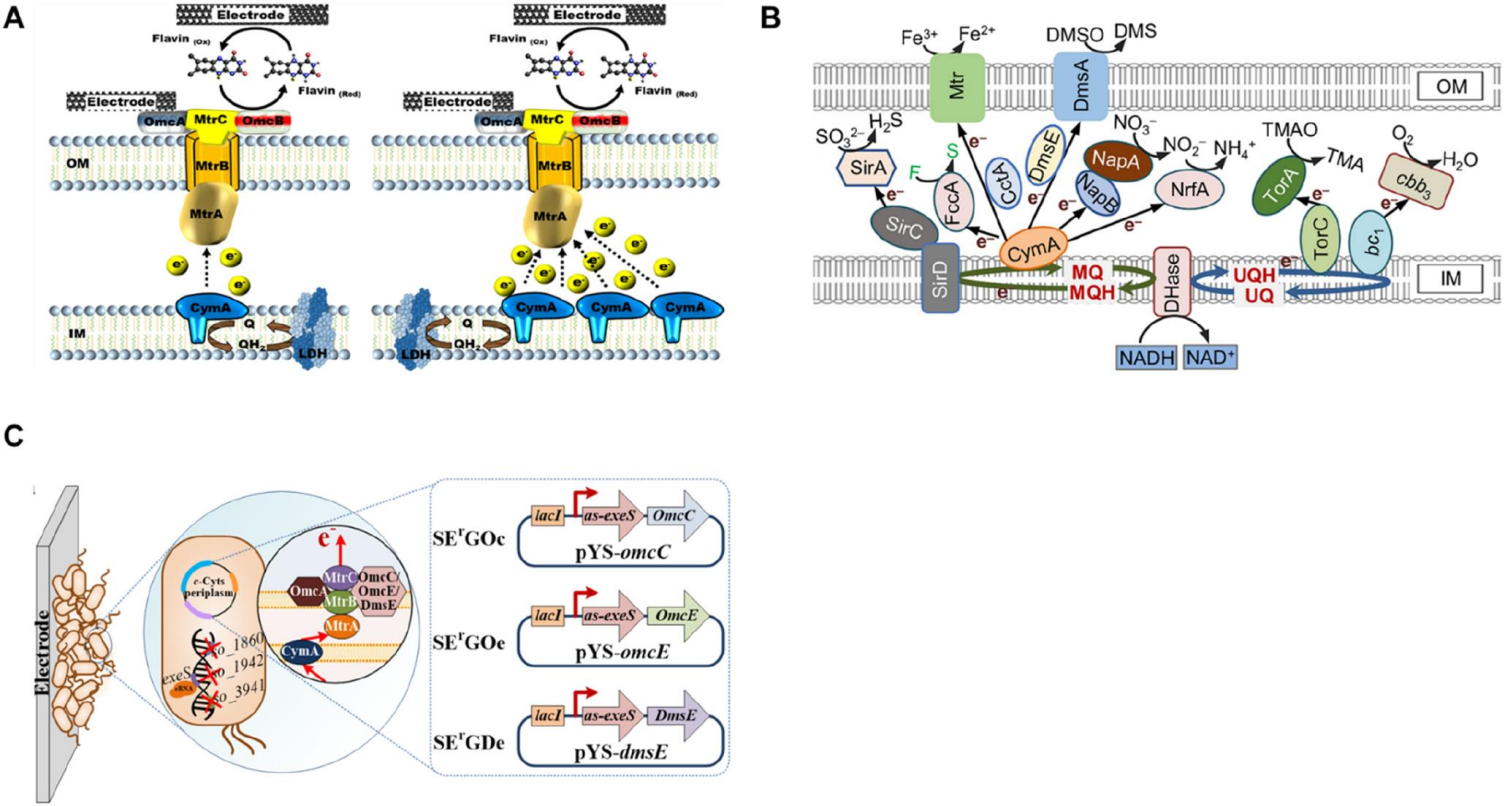
Engineering the expression of cytochromes. (A) Overexpressing *cymA* gene to enhance EET. Left, scheme of native cytochrome CymA expression. Right, scheme of enhanced expression of cytochrome CymA [74]. (B) Optimization of periplasmic cytochrome expression network. Knockdown of cytochromes NapB, FccA, and TsdB and overexpression of CctA enhance electron transfer capacity in *Shewanella oneidensis* [75]. (C) Overexpression of cytochrome OmcE, DmsE, and OmcC, respectively, in engineered strain to enhance electron transfer capacity. Cytochromes OmcC and OmcE are from *Geobacter sulfurreducens*, DmsE is from *Sideroxydans lithotrophicus.* Engineered strain were constructed by deleting *SO1860* encoding extracellular polysaccharide and *SO1942 and SO3941* encoding c‐di‐CMP as well as inhibiting nucleic acid degradation [76]. The reproduction with permission. EET, extracellular electron transfer.

Electron capture by inner membrane cytochrome CymA is the first step in mediating electron transfer to outer membrane redox proteins (e.g., MtrA, OmcA, and OmcB). CymA overexpression is considered a viable strategy to improve the electrical performance of MFCs due to its ability to distribute respiratory electrons and balance the oxidation‐reduction state [73]. As shown in Figure 4A, by overexpressing *cymA* gene in *S. oneidensis* MR‐1, the engineered strain obtained a higher output voltage (340 mV) and power density (0.13 mW) [74]. In another study, Jensen et al. verified the function of cymA protein in electron transport by expressing CymA, MtrCAB and the CymA‐MtrCAB complexes in *Escherichia coli*, respectively, and showed that the extracellular electron transport capacity of *E. coli* was significantly improved only when CymA‐MtrCAB was expressed simultaneously [77]. These results suggest that increased inner membrane cytochrome expression levels can elevate intracellular electron output flux and promote EET.

#### 3.3.3 Enhancing the expression level of periplasmic cytochromes

EAMs with a large periplasmic span (e.g., ∼235 Å in *S. oneidensis* MR‐1) have difficulties in direct electron transfer via inner and outer membrane cytochromes. Therefore, free multiheme *c*‐Cyt in the periplasm is required to assist the Mtr system acquiring electrons from CymA and transfer to the extracellular electron acceptors. Current studies demonstrated that *S. oneidensis* MR‐1 contains 27 periplasmic cytochromes, accounting for approximately 64% of the total cytochrome content, with the most abundant cytochromes being ScyA, FccA, and CctA, respectively [78]. Whether all of the abundant periplasmic cytochromes are involved in electron transport has not been demonstrated. Inspired by this, Delgado et al. used the *cctA* gene encoding the STC protein to sequentially *in situ* replace the *nrfA*, *ccpA*, *napA*, *napB* and *fccA* genes encoding periplasmic cytochrome proteins in the genome of *S. oneidensis* MR‐1, and successfully obtained five mutant strains. Electrochemical analysis revealed that the EET capacity could increase gradually with increasing the copy number of the *cctA* gene. Among them, the four *S. oneidensis* mutants (*ΔnrfA*::*cctA;* Δ*ccpA*::*cctA;* Δ*napA*::*cctA;* and *napB*::*cctA*) exhibited the highest EET capacity, with a 23% increase in current density compared to the wild‐type strain *S. oneidensis* MR‐1 [79]. This suggested that the abundant periplasmic cytochrome network is not fully involved in the electron transfer, yet how this network affects electron transfer remains elusive.

To address this issue, Sun et al. systematically analyzed the effect of single overexpression or deletion of the periplasmic *c*‐Cyts network components on the EET efficiency (Figure 4B). The data showed that overexpression of *cctA* accelerated the ability of electron transport from CymA to MtrCAB, whereas overexpression of *napB*, *fccA*, and *tsdB* produced opposite results. Subsequently, it was found that knockdown of *napB*, *fccA* and *tsdB* genes along with overexpression of *cctA* could further improve the electron transfer ability, obtaining a maximum power density of 436.5 mW m^−2^, which was ∼3.62‐fold higher compared to the wild‐type *S. oneidensis* MR‐1 [75]. These results imply that optimization of periplasmic *c*‐Cyts composition is an important strategy to improve the EET efficiency.

#### 3.3.4 Enhancing the expression of outer membrane cytochromes

Jensen et al. expressed the Mtr pathway in *E. coli* and successfully transferred intracellular electrons along a molecularly defined route to extracellular inorganic receptors. Compared to the controlled *E. coli* strain, introducing the Mtr pathway resulted in ∼8‐ and ∼4‐fold higher metal ions and solid metal oxides reduction rate, respectively [80]. Subsequent studies found that the introduced CymA‐Mtr enabled it to couple with the central metabolism of *E. coli* and alter the metabolite spectrum while changing the intracellular redox state [81]. To investigate the role of outer membrane cytochromes in electron transfer, by knocking out the *omcA* and *mtrC* genes in *S. oneidensis* MR‐1, Jing et al. found that OmcA was able to increase the attraction between the bacteria and electrode by 60%, while MtrC could increase the cell‐electrode contact area by more than twofold [82]. This study showed that OmcA and MtrC work together to improve the contact between cells and electrodes, thereby maximizing the efficiency of interfacial electron transfer. The outer membrane cytochrome OmcS is also an important conductive cytochrome protein, capable of transferring electrons directly from the cell to the terminal electron acceptor. Sekar et al. first heterologously expressed *omcS* gene from *G. sulfurreducens* PCA in the cyanobacterium *Synechococcus elongatus* PCC 7942 and successfully enhanced the electroactivity of the engineered strain, yielding a maximum power density of 300 mW m^−2^ [83]. To further improve biofilm electroactivity, Tang et al. heterologously expressed three outer membrane cytochrome proteins, OmcC, OmcE, and MtoA, respectively, and showed that the expression of the cytochromes OmcC and OmcE resulted in a greater improvement of electron transfer capacity, reaching 1002 ± 19.5 mW m^−2^, 843 ± 17.7 mW m^−2^, ∼1.2‐ and ∼0.93‐fold higher than that of the control strain (781 ± 16.5 mW m^−2^), respectively (Figure 4C) [76]. Overall, these studies suggested that enhancing cytochrome expression levels based on synthetic biology strategies is an important means to improve the electron transport capacity of EAMs.

## 4 STRUCTURE, FUNCTION, CONDUCTIVITY MECHANISM, AND ENGINEERING STRATEGIES OF CONDUCTIVE NANOWIRES

Conducive nanowires, including e‐pili and cytochrome nanowires, are essential for mediating long‐range electron transfer in EAMs. Many progresses have been made in elucidating the structure and mechanism of nanowires, which provide new potential for constructing novel conductive nanowires. In this section, the structure and composition of conductive nanowires, the mechanism of electron transfer, and the engineering strategies are reviewed.

### 4.1 Structure and function of conductive nanowires

Microbial conductive nanowires found on the surface of *G. sulfurreducens* [84] showed long‐distance electron transport capability [85, 86]. Similar conductive nanowires were also observed in *Synechocystis* PCC6803, *Peloomaculum thermopropionicum*, and *Lysinibacillus varians* GY32 [87, 88, 89]. Up to now, conducting nanowires have evolved for several generations during the evolution of microorganisms [90, 91, 92]. Except for playing an essential role in anaerobic respiration [90, 93, 94], conducting nanowires can also provide direct channels for intercellular information exchange [94, 95]. Here, we classify the conducting nanowires into cytochrome‐based nanowires (such as OmcS, OmcZ, and OmcE) and pili‐based nanowires (e‐pili) based on their structures and functions. The details of these nanowires are also listed in Table 1.

**TABLE 1:** Classification, structure and conductivity of conductive nanowires.

	Protein nanowire source	Nanowire diameter	Conductivity of nanowires	References
Pili‐based nanowires				
pilA‐*Gs*	*Geobacter sulfurreducens*	3 nm	51 mS/cm	[96, 97]
pilA‐*Gm*	*Geobacter metallireducens*	3 nm	277 S/cm	[98]
pilA‐*Pa*	*Pseudomonas aeruginosa*	5 nm	<*G. sulfurreducens*	[99]
pilA‐*Sa*	*Syntrophus aciditrophicus*	4 nm	∼*G. sulfurreducens*	[100]
pilA‐Mh	*Methanospirillum hungatei*	10 nm	>*G. sulfurreducens*	[101]
Cytochrome‐based nanowires				
OmcS	*G. sulfurreducens*	35 Å	30 mS/cm	[102]
OmcZ	*G. sulfurreducens*	23 Å	30 S/cm	[103]
OmcE	*G. sulfurreducens*	40 Å	‐	[104]

#### 4.1.1 Pili‐based nanowires

Conductive pili (e‐pili) from *G. sulfurreducens* [96] and *Geobacter metallireducens* [98] are the most studied type of conductive nanowires. Typically, *G. sulfurreducens* e‐pili is assembled from 61 amino acid pilin monomers with a diameter of 3 nm [93], which is thinner than other microorganisms, possibly due to a short and flexible C‐terminal random coiled segment [84, 105]. Studies have reported that the conductivity of individual e‐pili from *G. sulfurreducens* was 51 mS cm^‐1^ at pH = 7, and in another study showed its conductivity to be 1.4–4.3 S cm^−1^ [97]. While, the e‐pili of *G. metallireducens* exhibited higher electrical conductivity (277 S cm^−1^) due to their higher abundance of aromatic amino acids [98]. The conductivity of native e‐pili for other microorganisms has not been reported yet. Some evidence suggested that the abundance and location of aromatic amino acids in e‐pili are critical for conductivity. Increasing the amount of aromatic amino acids in e‐pili can improve conductivity, while e‐pili with lower abundance of aromatic amino acids, or large non‐aromatic gaps in the amino acid sequence, exhibits lower conductivity [92, 98, 106, 107, 108, 109, 110, 111]. During e‐pili assembly, the tight packing of aromatic amino acids forms an aromatic‐to‐aromatic electron transport pathway, which may be the mechanism for electron transport along the length direction.

#### 4.1.2 Cytochrome‐based nanowires

Recent studies showed that *G. sulfurreducens* is capable of forming cytochrome nanowires, such as 4 nm‐diameter nanowires composed of hexaheme *c*‐type cytochrome OmcS [102] or 2.5 nm‐diameter nanowires composed of octaheme *c*‐type cytochrome OmcZ [103]. As revealed by cryo‐electron microscopy, the distance between heme stacks in OmcS nanowires is 3.5–6 Å. This parallel stacking of heme and the axial co‐ordination of heme by histidine from adjacent subunits allows the conductivity of individual OmcS to reach 30 mS cm^‐1^. Surprisingly, under electric field stimulation, *G. sulfurreducens* can produce highly conductive OmcZ nanowires with a 1000‐fold higher conductivity (30 S cm^−1^), which is caused by the tight stacked heme [1].

Another thinner OmcE cytochrome nanowire was recently identified on *G. sulfurreducens*. Although the OmcE and OmcS subunits showed no sequence or structural similarity, they have a conserved heme packing arrangement, in which the heme is coordinated by histidines in adjacent subunits [104]. The abundance of cytochrome nanowires varies highly depending on the growth conditions of *G. sulfurreducens*. In some cases, pilin or cytochrome nanowire dominate; in other cases, there is a mixture of cytochrome‐based and pilin‐based nanowires. And the seamless polymerization of cytochromes and the stringent growth conditions make it a great challenge to engineer cytochrome nanowires for practical applications.

### 4.2 Electron transfer mechanism of conductive nanowires

EAMs can achieve long‐distance electron transfer to terminal electron acceptors using e‐pili with electron conductivity comparable to that of conducting polymer nanowires [112, 113]. Indeed, several strains of *G. sulfurreducens* conductive nanowires have been shown to have long‐range electrical conductivity [114].

However, the mechanism of electron transfer in e‐pili is still hotly debated. One view is the delocalized charge transfer theory, which suggests that the conductivity of nanowires is mainly due to the overlap of π–π bond orbitals of aromatic amino acids, leading to the charge transfer on the nanowires in a delocalized pattern [115]. It is notable that closely aligned aromatic residues do not guarantee π–π stacking [116]. In semi‐crystalline conducting polymers, there is a core of aromatic aggregates that allows for rapid intra‐chain electron transfer, with high mobility as electrons hop between delocalized regions [117]. The idea is that while aromatic and charged residues act as electron traps, electrons can jump between peptide backbone carbons in multiple steps [118, 119]. Rather than acting as electron sinks, these residues allow for the delocalization leading to rapid electron transfer between proteins. Initial studies by Malvankar et al. using X‐ray diffraction, and report peaks can be explained as the dense aromatic packing of phenyl rings in e‐pili, indicating that the π‐orbitals overlap and the charge delocalization [115].

Another view is the electron hopping theory, which suggests the electrical conductivity of nanowires is mainly due to the seamless stacking of heme structures to form micron‐sized filaments that provide a continuous path for electron hopping. Taking OmcZ and OmcS nanowires as an example, the latest structure of cytochrome nanowires shows that OmcZ contains ∼12% helices and ∼28% β‐chains and turns, higher than the ∼22% β‐chains and turns found in OmcS. The OmcZ subunit has a compact linear structure with a smaller diameter (∼23 Å) than the OmcS nanowires (∼35 Å) [103]. Due to its compactness, the hemes in OmcZ nanowires are tightly stacked with edge‐to‐edge distances substantially smaller than those in OmcS nanowires, which may explain the higher conductivity of OmcZ.

In all, although significant progress has been made in experiments and computations, the conflicting conclusions have been drawn in the literature on the rationality of the sole mechanism for conductivity in *G. sulfurreducens* pili. In fact, there may be transient “metallic‐like” regions or partial hopping regions, just like the disorder exists in conductive polymers, the dynamic coupling of these structures can even promote conductivity [120].

### 4.3 Engineering strategies to enhance conductivity of nanowires

Enhancing the electrical conductivity of nanowires by rational design of the amino acid sequence of proteins could facilitate bio‐electrochemical applications. However, the practical engineering modifications of nanowires are difficult due to their naturally formed precise arrangement structure [102, 121]. Recent research work on conducting nanowires has focused on heterologous construction of e‐pili, regulation of the aromatic amino acid content of the pili monomer protein (pilA), and design of peptide structures to modify the pili monomer, which enhanced the conductivity of e‐pili.

#### 4.3.1 Regulation of composition of pilin monomers

**FIGURE 5:**
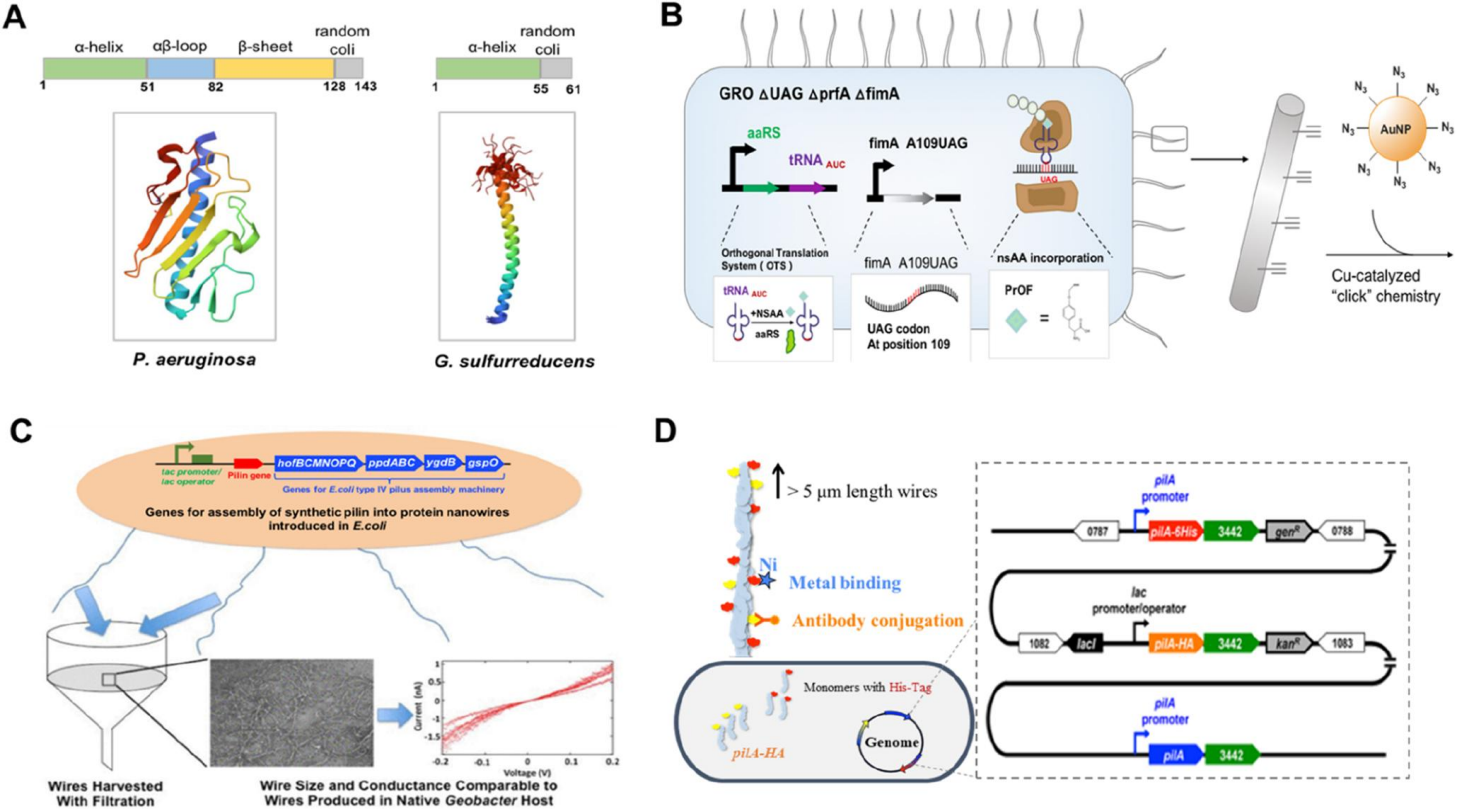
Engineering e‐pili. (A) Tertiary structure and sequence diagram of PaPilA and GsPilA. Expression and effects of GsPilA and truncated PaPilA in *Pseudomonas aeruginosa* PAO1 and Δ*pilA* mutant [122]. (B) Engineering design for electronic conductivity of pili nanowires. Construction of nsAAs‐encoded modified protein nanowires coupled with gold nanoparticles to enhance the pilus conductivity [123]. (C) Construction of e‐pili synthesis system in *Escherichia coli*. Introducing a plasmid that contained an inducible operon with *E. coli* genes for *Geobacter sulfurreducens* e‐pili biogenesis machinery and genetic manipulation [9]. (D) Decorating the outer surface of e‐pili with peptides. Construction of e‐pili nanowires with a six‐histidine “His‐Tag” or both the His‐Tag and a nine‐peptide “HA‐Tag” [10]. The reproduction with permission.

To improve conductivity of e‐pili, Tan et al. first heterologously expressed the *pilA* gene from *G. metallireducens* into *G. sulfurreducens*. The conductivity of engineered e‐pili reached up to 277 S cm^−1^ (at pH = 7), which was 5000‐fold higher than the conductivity of e‐pili from *G. sulfurreducens* [98]. Similarly, by heterologous expressing the gene for the *pilA* pilin monomer of *G. sulfurreducens* into *Pseudomonas aeruginosa*, the pili with a diameter of 3.2 ± 0.2 nm were produced [122], compared with 4.8 ± 0.6 nm in wild‐type of *P. aeruginosa*, and their conductivity was 20‐fold higher than that of wild type. Liu et al. obtained PaPili_1‐61_M_3_ with high conductivity by truncating the C‐terminus of the pilA structure and increasing the content of aromatic amino acids in the N‐terminal α‐helical region to reconstruct e‐pili of *P. aeruginosa* (Figure 5A), which greatly improved the current output [122].

Recently, through a combined approach of structure elucidation, computational simulation to identify amino acid targets and targeted mutation screening, researchers have incorporated tryptophan into pili to increase the electrical conductivity of individual pilus by more than 80‐fold, and genetically encoded modification of the nonstandard amino acid propargyl‐oxy‐phenylalanine (PrOF) by redesigning it in the *E. coli* genome enabled the pili to act as a functional scaffold for precise and specific binding of gold nanoparticles, then form ordered organic‐inorganic hybrid biomaterials, which improved their electrical conductivity by 170‐fold (Figure 5B) [123]. These results suggested that e‐pili can be significantly improved by varying its aromatic amino acid content.

#### 4.3.2 Design of peptide structure to modify pili monome

Since *Geobacter* culture requires a strictly anaerobic environment, it is difficult to perform genetic manipulation and obtain large amounts of e‐pili for diverse applications. Lovley et al. used *E. coli* as a chassis strain and reconstructed the GspilA synthesis system, knocking out its own *pilA* gene to obtain the same e‐pili structure and conductivity as those expressed in *G. sulfurreducens*. This method provided a new way to manufacture novel e‐pili on a large scale under conventional culture conditions (Figure 5C) [9, 124].

In addition to the design of the pili structure to enhance electrical conductivity, use of peptides to modify the pilin monomer structure can increase the range of pili applications. Ueki et al. designed short peptide tags fused to the C‐terminus of pilA, which can produce specific adhesion of e‐pili without affecting its electrical conductivity. The synthesized gene fragments contain pilA‐6His and pilA‐HA, resulting in e‐pili with “His‐Tag” and peptide “HA‐Tag” (Figure 5D), which can bind metals and corresponding antibodies when exposed to the extracellular surface [10]. This simplicity of e‐pili production made it possible to develop many functions, for example, conductive nanowires can be used to construct biosensors [125, 126]. In all, although the development of e‐pili is still in its infancy, the strong advantages and large‐scale production of e‐pili made it a sustainable electronic material.

## 5 CONCLUSIONS AND PERSPECTIVE

Microbial electrochemical systems driven by EAMs have shown promising applications in clean energy development, environmental monitoring, organic catalysis and biomedicine. In this review, we first reviewed the quantitative kinetics of electron transfer through mathematical modeling, and performed analysis of biochemical reaction processes in EET. These quantitative analyses were important for guiding the rational design and engineering of EAMs. We then systematically reviewed the structures, functions, conductivity mechanisms, and engineering strategies to redesign the conductive cytochromes and nanowires in EAMs, which will further accelerate the development of EAMs for microbial electrochemical systems in biomanufacturing and biocatalysts for sustainable production of green energy and chemicals.

At present, there are still limitations for the mechanistic resolution and engineering modification of EAMs. Below, future research directions could be considered in the following aspects:

1. In‐depth exploration of electron transfer mechanisms. As new EAMs are being discovered, new electron transfer mechanisms are being elucidated. However, precisely monitoring electron transfer is challenging since electron transfer between microorganisms is difficult to measure directly. Therefore, quantitative assessment of electron transfer kinetics through mathematical modeling is an important approach to explore how microorganisms communicate, grow, and develop. (i) Although EET‐based quantitative analysis is still in its early stages, more quantitative techniques, such as isothermal calorimetry, surface plasmon resonance, interdigitated electrode arrays, double step chronoamperometry and differential pulse voltammetry (DPV) can be developed in the future to quantify protein interactions and the thermodynamics and kinetics of electron transfer within proteins [127, 128]. (ii) Rapid advances in machine learning [67] and artificial intelligence [129] may have great impact on the quantification of electron transfer. Large‐scale data obtained through mathematical model calculations offer the possibility for machine learning to predict the molecular behaviors of electrons in cytochromes, visualize electron transfer trajectories, quantify electron transfer kinetics, and design efficient electron transfer pathways.
2. Reconstitution of cytochromes and conductive nanowires. Previous quantification of electron transfer kinetics provided an important theoretical basis for the engineering modification of cytochromes and conductive nanowires‐mediated electron transfer pathways. However, the spatial structure of the cytochromes and heme distribution mechanisms are still not elucidated, limiting the potential to engineer cytochromes or conductive nanowires. (i) Future research could employ machine learning to predict molecular protein conformation, assess the adaptive mechanism of cytochromes and heme, and optimize the binding sites and spatial arrangement between cytochrome complexes to enhance electron transfer [68]. It is also possible to combine genome informatics, transcriptomics and proteomics technologies to identify key proteins involved in nanowire assembly and secretion processes, and to design aromatic amino acid‐rich conductive nanowires rationally with the help of machine learning to fundamentally optimize the conductivity of conductive nanowires. (ii) Based on the understanding of cytochrome protein structures, self‐assembled peptides containing heme binding sites can be designed in the future, allowing them to bind to heme and achieve electron transport at the micron scale [130]. (iii) Moreover, constructing highly conductive biohybrids based on nanomaterials or polymers also shows great potential in facilitating electron transfer. For example, nanomaterials (e.g. Au [131], polymers [132], CdS [133, 134] and InP [135]) that are superior to cytochromes and conductive nanowires for high‐speed electron transport can be integrated as artificial nanowires into engineered EAMs. These integrated systems will bring new possibilities for bioelectricity production, CO_2_ fixation and synthesis of value‐added chemicals [136, 137].

## AUTHOR CONTRIBUTIONS

Junqi Zhang and Zixuan You drafted the manuscript; Dingyuan Liu, Rui Tang, and Chao Zhao collated the pictures and tables, Yingxiu Cao, Feng Li, Hao Song critically revised the manuscript. All authors read and approved the manuscript.

## CONFLICT OF INTEREST STATEMENT

The authors Junqi Zhang, Zixuan You, Dingyuan Liu, Rui Tang, Chao Zhao, Yingxiu Cao, Feng Li, and Hao Song declare no conflicts of interest.

## ETHICS STATEMENT

This is a review article and does not involve any research related to human or animal subjects.
